# Natural Killer Cell Inhibitory Receptor Expression in Humans and Mice: A Closer Look

**DOI:** 10.3389/fimmu.2013.00065

**Published:** 2013-03-26

**Authors:** Michal Sternberg-Simon, Petter Brodin, Yishai Pickman, Björn Önfelt, Klas Kärre, Karl-Johan Malmberg, Petter Höglund, Ramit Mehr

**Affiliations:** ^1^The Mina and Everard Goodman Faculty of Life Sciences, Bar-Ilan UniversityRamat-Gan, Israel; ^2^Department of Medicine Huddinge, Center for Hematology and Regenerative Medicine, Karolinska InstitutetStockholm, Sweden; ^3^Department of Microbiology Tumor and Cell Biology, Karolinska InstitutetStockholm, Sweden; ^4^Department of Applied Physics, KTH – Royal Institute of TechnologyStockholm, Sweden; ^5^Department of Medicine Huddinge, Center for Infectious Medicine, Karolinska InstitutetStockholm, Sweden; ^6^Institute for Cancer Research, Oslo University HospitalOslo, Norway; ^7^Institute for Cancer Research, Institute of Clinical Medicine, University of OsloOslo, Norway

**Keywords:** killer immunoglobulin-like receptor, Ly49, MHC class I, product rule, repertoire

## Abstract

The Natural Killer (NK) cell population is composed of subsets of varying sizes expressing different combinations of inhibitory receptors for MHC class I molecules. Genes within the NK gene complex, including the inhibitory receptors themselves, seem to be the primary intrinsic regulators of inhibitory receptor expression, but the MHC class I background is an additional Modulating factor. In this paper, we have performed a parallel study of the inhibitory receptor repertoire in inbred mice of the C57Bl/6 background and in a cohort of 44 humans. Deviations of subset frequencies from the “product rule (PR),” i.e., differences between observed and expected frequencies of NK cells, were used to identify MHC-independent and MHC-dependent control of receptor expression frequencies. Some deviations from the PR were similar in mice and humans, such as the decreased presence of NK cell subset lacking inhibitory receptors. Others were different, including a role for NKG2A in determining over- or under-representation of specific subsets in humans but not in mice. Thus, while human and murine inhibitory receptor repertoires differed in details, there may also be shared principles governing NK cell repertoire formation in these two species.

## Introduction

The activity of natural killer (NK) cells is controlled by balancing inputs from activating and inhibitory receptors. The most important ligands for inhibitory receptors are MHC class I molecules. Because normal cells express high levels of MHC class I, they are most often protected from NK cell killing. In contrast, target cells expressing downregulated levels of MHC class I are seen as “missing self” and killed (Ljunggren and Karre, 1990). Interactions between inhibitory receptors and MHC class I also affect NK cell development and tunes the responsiveness of the NK cell in an “education” process (Hoglund and Brodin, 2010). NK cells sense MHC class I in two ways: by direct recognition of individual MHC class Ia alleles or by indirect recognition of an MHC class Ib allele complexed with peptides derived from class Ia molecules. Both pathways are represented in mice and in humans, suggesting that strong selective forces have acted to maintain their parallel use. Inhibitory recognition of MHC class Ib molecules is performed by the NKG2A receptor in mice and humans, where it recognizes Qa-1^b^ and HLA-E, respectively. Mice and humans differ, however, in the types of inhibitory receptors they use for direct recognition of MHC class Ia molecules. While mice use lectin-like receptors of the Ly49 family, human NK cells use killer immunoglobulin-like receptors (KIR). In humans, KIR immunogenetics have been coupled to autoimmune diseases (Martin et al., 2002), infections (Martin et al., 2002; Khakoo et al., 2004; Pelak et al., 2011), and outcomes in transplantation (Ruggeri et al., 2002; Cooley et al., 2009; Venstrom et al., 2012), suggesting that KIR genes take part in immunological fine-tuning. KIR-HLA interactions have also been proposed to influence NK cell-mediated remodeling of spiral arteries in the placenta affecting the outcome of pregnancy (Hiby et al., 2004). Altogether, these associations imply KIR genes as major regulators of human biology.

Despite the major structural differences between KIR and Ly49 proteins, the central properties of the two gene families are remarkably similar. First, both families are polygenic, containing between 15 and 20 genes each (Makrigiannis and Anderson, 2000; Moesta and Parham, 2012) and individuals (or mouse strains) vary with respect to which of these genes they carry. Secondly, both families show polymorphism in individual genes. Thirdly, the expression of the gene product at the cell surface is stochastic, giving rise to highly variegated repertoires (Andersson et al., 2009; Brodin and Hoglund, 2008). The extent of receptor polymorphism is most studied in humans, and new alleles for individual KIR genes are discovered continuously (Jiang et al., 2012). Polymorphisms in mice are less well characterized since focus so far has been on a few inbred mouse strains: C57Bl/6 (B6), Balb/C, NOD, and 129 (Makrigiannis and Anderson, 2000; Patel et al., 2010). This comparison has reveled major differences in terms of gene content, but has also shown sequence differences suggesting polymorphisms in single genes. From those data, a major diversity of NK cell receptors can be inferred also in mice, but until the NK gene complex in wild rodents from different locations has been analyzed, the extent of genetic variation in the mouse remains unknown.

The magnitude of sequence polymorphisms in individual KIR genes is extensive (Jiang et al., 2012). One would think that this polymorphism has developed to match the rapid evolution of individual HLA class I alleles, making room for a large variation in individual KIR/HLA-interaction patterns. Surprisingly, however, the specificity of KIR/HLA interactions seems to be rather conserved at the level of HLA recognition, and only four major HLA recognition motifs have been identified, each recognized by one or several KIRs (Moretta and Moretta, 2004). The role of KIR allelic polymorphism in this limited world of ligands is therefore unclear. It may affect interaction strength more than overall ligand specificity (Hilton et al., 2012), and could also influence other properties of KIR genes, such as expression patterns, that may be unrelated to the HLA binding capacity of the receptors (Yawata et al., 2008). KIR and Ly49 receptors bind MHC class Ia molecules in a different manner (Achour et al., 1998; Chen et al., 2009). Data from mice nevertheless suggest that Ly49 recognition may also be flexible in terms of the number of ligands that are recognized by each Ly49 receptor (Hanke et al., 1999; Johansson et al., 2009). Because of the limited number of individual Ly49 and MHC class I alleles that have been studied, it is not possible to know if Ly49 recognition follows a similar recognition rule as KIR receptors, or if recognition is directed against unique residues in individual MHC class I alleles.

A remarkable feature of KIR and Ly49 receptors is the stochastic and variegated expression patterns that are seen in individual mouse and human NK cells. The genetic regulation of this process is only partly understood, but the outcome is a “repertoire” of NK cells expressing anywhere from zero to five or six inhibitory receptors (Yawata et al., 2008; Andersson et al., 2009; Schonberg et al., 2011b; Brodin et al., 2012). Because NK cell repertoires differ between individuals, it has been suggested that KIR gene polymorphisms, gene copy number content, epigenetic factors, and environmental cues cooperate in repertoire formation (Uhrberg, 2005; Pascal et al., 2006). The presence of MHC class I ligands for inhibitory receptors also affect the frequencies of individual NK cell subsets, which has been interpreted in terms of an “adaptation” process to ensure optimal “missing self” recognition in each individual (Hoglund and Brodin, 2010). The evidence for this is strongest in mice, where direct comparisons of the inhibitory receptor repertoires in MHC class I-deficient and sufficient mice have been made (Held et al., 1996; Salcedo et al., 1998; Fahlen et al., 2001; Brodin et al., 2009, 2012). In humans, links between the presence of self HLA ligands and expansion or contraction of certain NK cell subsets have been difficult to demonstrate (Shilling et al., 2002; Andersson et al., 2009). High resolution KIR phenotyping has however shown effects of HLA on the KIR repertoire (Yawata et al., 2006; Yu et al., 2007). Our recent data suggest that KIR ligands do have a clear effect on the KIR repertoire but only in individuals infected by CMV (Beziat et al., 2013).

In this article, we will discuss the formation of murine and human inhibitory receptor repertoires. We will first describe the repertoire in B6 mice, and examine how the observed repertoires differ from the expected repertories under the assumption that repertoire composition is completely random. Using this analysis, we identified both MHC-independent and dependent effects of the repertoire. We will finally compare the murine repertoire of one single strain with that of a cohort of humans homozygous for the group A KIR haplotype.

## Materials and Methods

### Mice

Inbred mice of the B6 genetic background were used. B6 mice lacking functional MHC class I heavy chain genes (B6.K^b^D^b−/−^ mice) were a kind gift from Francois Lemonnier, Institut Pasteur, France. B6.K^b^D^b−/−^ mice were obtained by crossing mice made deficient for the K^b^ and D^b^ genes respectively (called B6.K^b−/−^ and B6.D^b−/−^ mice) as described previously (Perarnau et al., 1999; Lemonnier, 2002). To generate mice expressing D^d^ as the only MHC class Ia allele, H2D^d^ transgenic B6 mice (Hoglund et al., 1988) were crossed to B6.K^b^D^b−/−^ mice, followed by further backcross of the progeny to B6.K^b^D^b−/−^ mice. Individuals homozygous for the K^b^ and D^b^ mutations and positive for the D^d^ transgene were identified using FACS analysis. A similar mating scheme was followed to generate mice expressing L^d^ alone starting with H2L^d^ transgenic B6 mice (Johansson et al., 2005, 2009). All animal experiments were performed in accordance with relevant institutional and national guidelines and regulations and conform to relevant regulatory standards. Ethical evaluations were performed by the animal ethics committee of northern Stockholm. Mice in each experiment were age (6–10 weeks) and sex matched. Both female and male mice were used.

### Human subjects

Experiments on human blood samples were approved by the regional ethics committee (Stockholm, Sweden, approval number 2006/229-31/3). Buffy coats were prepared from peripheral blood of healthy human donors (ages approximately 20–60 years old) and separated by density gradient centrifugation (Ficoll-Hypaque™, GE Healthcare Bio-Sciences AB, Uppsala, Sweden). The cohort of humans used in this study is the same as the one used in our previous study, and includes 44 individuals homozygous for the group A KIR haplotype (Andersson et al., 2009).

### Flow cytometry

Murine NK cells were isolated from the spleen and human NK cells from peripheral blood (Andersson et al., 2009; Brodin et al., 2012). The antibody panels and protocols for multi-color flow cytometry used for the repertoire studies in mice and humans have been described in detail (Fauriat et al., 2008; Andersson et al., 2009; Brodin et al., 2012). In brief, human NK cell receptor antibodies were KIR3DL1 (DX9), KIR2DL1/S1 (EB6), KIR2DL2/3/S2 (GL183), KIR3DL2 (DX31), and NKG2A (z199). For the mouse analysis, Ly49A (YE1/48), Ly49C (4LO3311), Ly49I (YLI-90), Ly49G2 (4D11), and NKG2A/C/E (20d5) antibodies were used. Gating strategies for the FACS data used here have been described (Fauriat et al., 2008; Brodin et al., 2012). In summary, murine NK cells were identified as NK1.1 + CD19−CD3− and human NK cells as CD56 + CD14−CD3−. All stainings in both species excluded dead cells and doublets. Receptor expression was evaluated on these gated subsets.

### Product rule calculations

The observed exclusive expression probabilities of combinations of zero to five receptors were calculated from the experimental data. In addition, we calculated the expected exclusive expression probabilities for these combinations of receptors, based on the overall expression frequency of each receptor in the data, and assuming that the expression follows the product rule (PR). Deviations of the observed from the expected frequencies of expression of receptor combinations are plotted as *log_2_ (observed/expected)*. In box plot figures, the box signifies upper and lower quartiles, and the median is represented by a black line within the box. Bottom and top whiskers represent the 5 and 95% values, respectively.

## Results

### The inhibitory receptor repertoire of B6 mice

**Figure 1:**
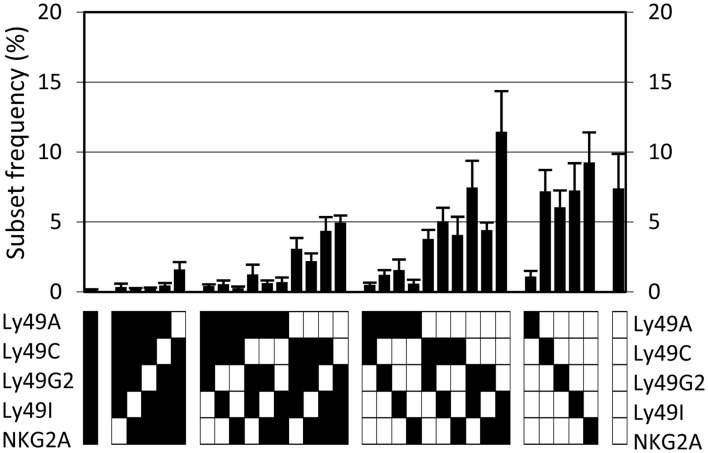
**The NK cell inhibitory receptor repertoire in B6 mice**. The observed frequency of each of 32 possible combinations, averaged for 37 mice, analyzed in six independent experiments. Error bars indicate the standard deviations. The lower panel indicates, for each combination, whether an individual receptor is expressed in this combination or not (black and white squares, respectively).

**Figure A1:**
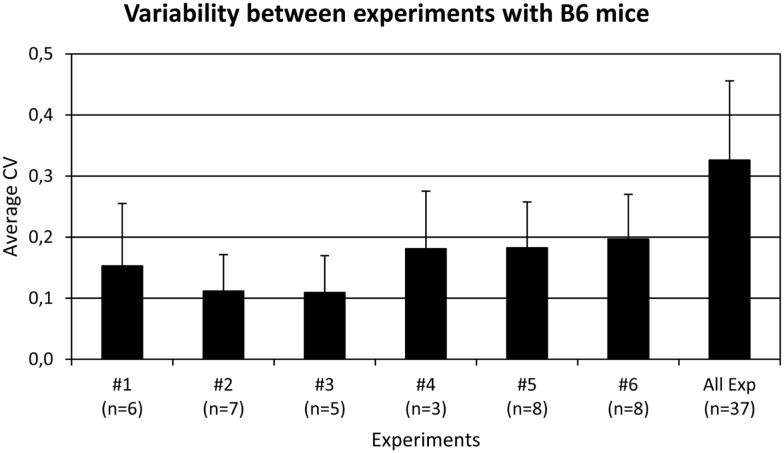
**Repertoire variations in each experiment separately (#1–#6) and in all mice together (All Exp)**. The average frequencies of each of the 32 NK cell subsets were first calculated in each group of mice. The coefficient of variance (CV, standard deviation/average) for each subset was determined and the average of all subsets in each group was plotted with the standard deviation. The number of mice in each experiment is indicated in parentheses. The CV’s were (from left to right): 015, 0.11, 0.109, 0.181, 0.182, 0.2, and 0.33. The higher the CV, the higher the variation is in the group.

A flow cytometry panel was used to simultaneously detect the frequency and expression levels of the following five inhibitory receptors on NK cells in B6 mice: Ly49A, Ly49C, Ly49G2, Ly49I, and NKG2A. Using this antibody panel, we determined the frequencies of NK cells expressing all possible combinations of these receptors, in total 32 subsets. Six experiments were performed, including 37 individual mice. All 32 possible subsets were present in the repertoire but in greatly varying frequencies (Figure 1). Of note, NK cells lacking all five receptors were among the most prevalent, reaching 7.4% in this analysis (Figure 1). The standard deviations were rather large for most subsets, which was surprising given the genetic homogeneity of this cohort of inbred mice (Figure 1). This variation was to a large extent due to inter-experimental variation of subset frequencies (Figure A1 in Appendix). However, there was also intra-experimental variation, of similar size in each experiment, that could reflect a biological variation, transient or permanent, in the expression of these inhibitory receptors.

### Observed subset frequencies deviate from those expected from product rule calculations

**Figure 2:**
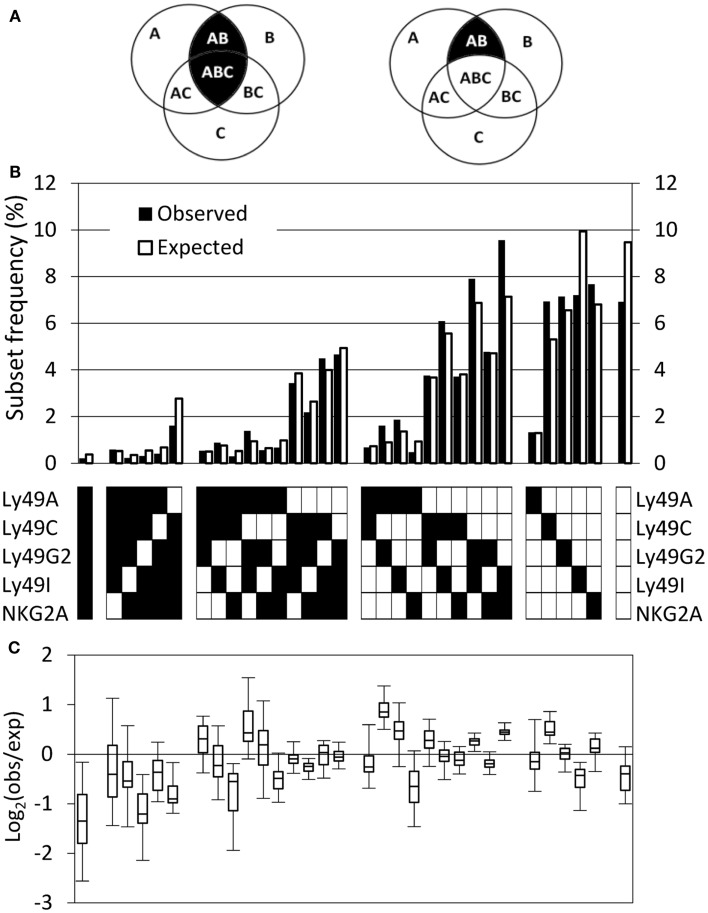
**The observed repertoire in B6 mice deviates from the product rule**. **(A)** The inclusive (left) and exclusive (right) calculation of the probability to express two out of three possible receptors. If the probability to express each of the receptors A, B, and C is *P*(A), *P*(B), and *P*(C), respectively, then the inclusive probability to express A and B is *P*(AB) = *P*(A) × *P*(B) and it includes all the cells that expresses both A and B, regardless to the expression of C, and therefore includes combinations AB and ABC. The exclusive probability to express A and B is *P*(AB) = *P*(A) × *P*(B) × [1−*P*(C)], and it includes only cells that express A and B, but do not express C. **(B)** The observed (black bars) and expected (white bars) repertoire of one representative mouse. **(C)** Deviations from the product rule in 37 B6 mice, shown as box plots of log_2_ (observed frequency/expected frequency). Negative values indicate that the respective combination is under-expressed, compared to the expected frequency, and positive values indicate over-expression.

In a model in which expression of individual genes is independently regulated, the fractions of cells expressing combinations of genes can be predicted using “PR” calculations. According to the PR, the joint probability of expression of two or more receptors is given by the product of the individual expression frequencies of these receptors (Mehr et al., 2012). Calculations can be performed in two ways: “inclusive” calculations examine the joint expression probability of a combination of receptors, regardless of other receptors expressed by the same cell. In contrast, “exclusive” calculations examine the joint expression probability of a combination of receptors, excluding cells that express any other receptors (Figure 2A). In this study, we have used the “exclusive” way of calculating the PR, since it predicts not only frequencies of NK cells expressing multiple receptors, but also subsets without receptors or with single receptors (Mehr et al., 2012). By comparing the observed and predicted expression frequency of each receptor combination, inter-dependencies between receptors and specific deviation in different genetic backgrounds, such as the comparison between MHC-deficient and B6 mice, can be revealed.

The observed and expected frequencies of all 32 Ly49 subsets in B6 mice are exemplified by a representative mouse (Figure 2B), as well as a summary of all mice in the cohort plotted as Log_2_ ratios between observed and expected frequencies (Figure 2C). Deviations were common and occurred in both directions, i.e., frequencies were both increased and decreased relative to the expected (Figure 2C). Examination of subsets expressing single Ly49 receptors for self MHC class I in B6 mice, Ly49C, Ly49I, or NKG2A (Brodin et al., 2012), did not reveal a consistent deviation pattern. In fact, these three subsets behaved completely differently; the Ly49C subset was over-represented, the Ly49I subset under-represented, and the NKG2A subset was close to unchanged. Subsets expressing two or three receptors showed deviations in both directions while subsets expressing no, four, or five receptors were in most cases less frequent than expected (Figure 2C).

### Product rule deviations are caused by a combination of MHC class I-dependent and independent events

**Figure 3:**
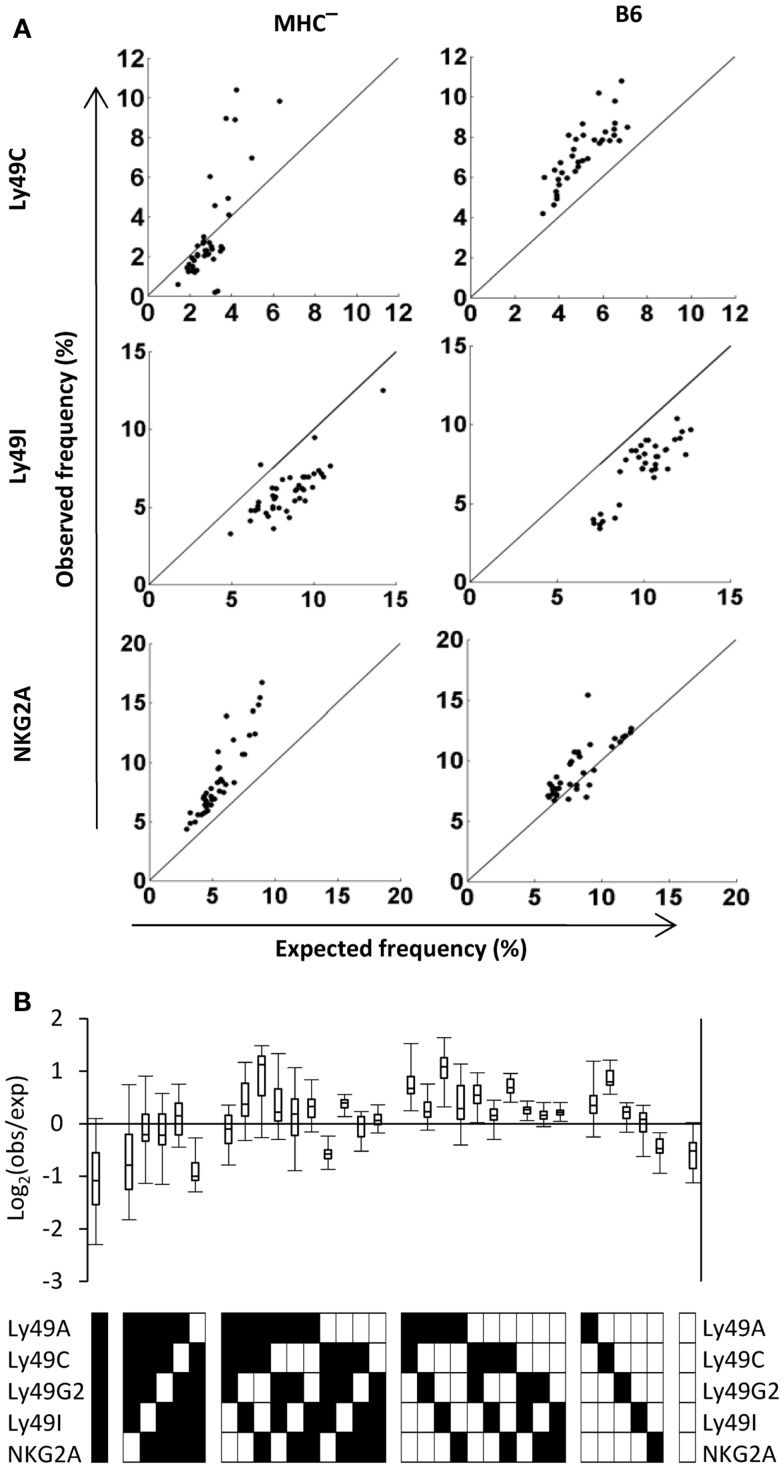
**Deviations from the product rule of single receptor combinations in B6 and MHC mice**. **(A)** The observed frequencies of NK cells expressing the indicated single receptor combination in 43 MHC and 37 B6 mice (left and right panel, respectively) are plotted against the expected frequency, under the product rule assumption. A perfect fit to the product rule is shown as a straight line. **(B)** Deviations from the product rule in 37 B6 mice, after subtracting, for each mouse and for each receptor combination, the average deviation measured in 43 MHC mice for the same combination.

To investigate to what extent PR deviations of the three subsets expressing single self-specific receptors were MHC class I-dependent, we performed the same analysis in a cohort of 43 mice lacking MHC class I molecules. Interestingly, deviations of these subsets were seen also in MHC-deficient mice (Figure 3A, left panel). For NK cells expressing only Ly49C, most MHC-deficient mice contained fewer such NK cells than predicted by the PR, but some mice showed an over-representation (Figure 3A, top left). This pattern was different from that in B6 mice, where all mice contained a higher frequency of these cells than expected (Figure 3A, top right). The pattern for Ly49I (under-representation) was very similar in MHC-deficient and in B6 mice (Figure 3, middle), while NKG2A single-positive NK cells were more frequent than expected in all MHC-deficient mice, yet showed a close to expected expression pattern in B6 mice (Figure 3A, bottom).

**Figure A2:**
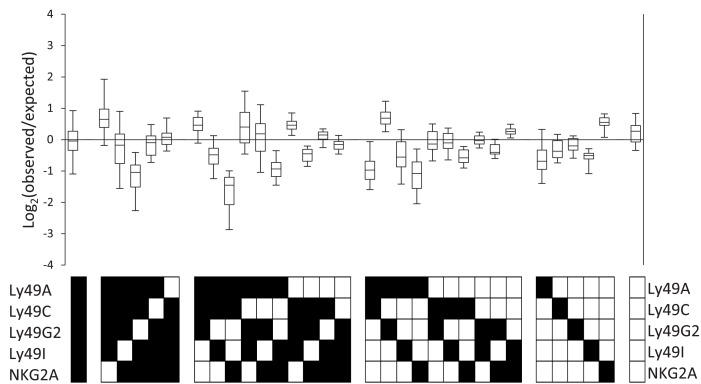
**Deviations from the product rule in 43 MHC-deficient mice, shown as box plots of log_2_ (observed frequency/expected frequency)**. Negative values indicate that the respective combination is under-expressed, compared to the expected frequency, and positive values indicate over-expression.

To reveal the total pattern of MHC class I-driven deviations from the PR of all 32 subsets in B6 mice, we subtracted the average deviation of each subset in the cohort of MHC-deficient mice (shown in Figure A2 in Appendix) from the deviation in each individual B6 repertoire. The resulting Log_2_ plot (Figure 3B) showed several differences compared to the unsubtracted B6 data (Figure 2C). A general effect was an increase in the number of subsets which were over-represented compared to expected, a pattern that was particularly clear for subsets expressing two receptors (Figure 3B). However, there were also subsets that went from being slightly over-represented to under-represented, such as NK cells expressing only NKG2A.

### Distinct repertoire perturbations in mice expressing single MHC class I alleles

**Figure 4:**
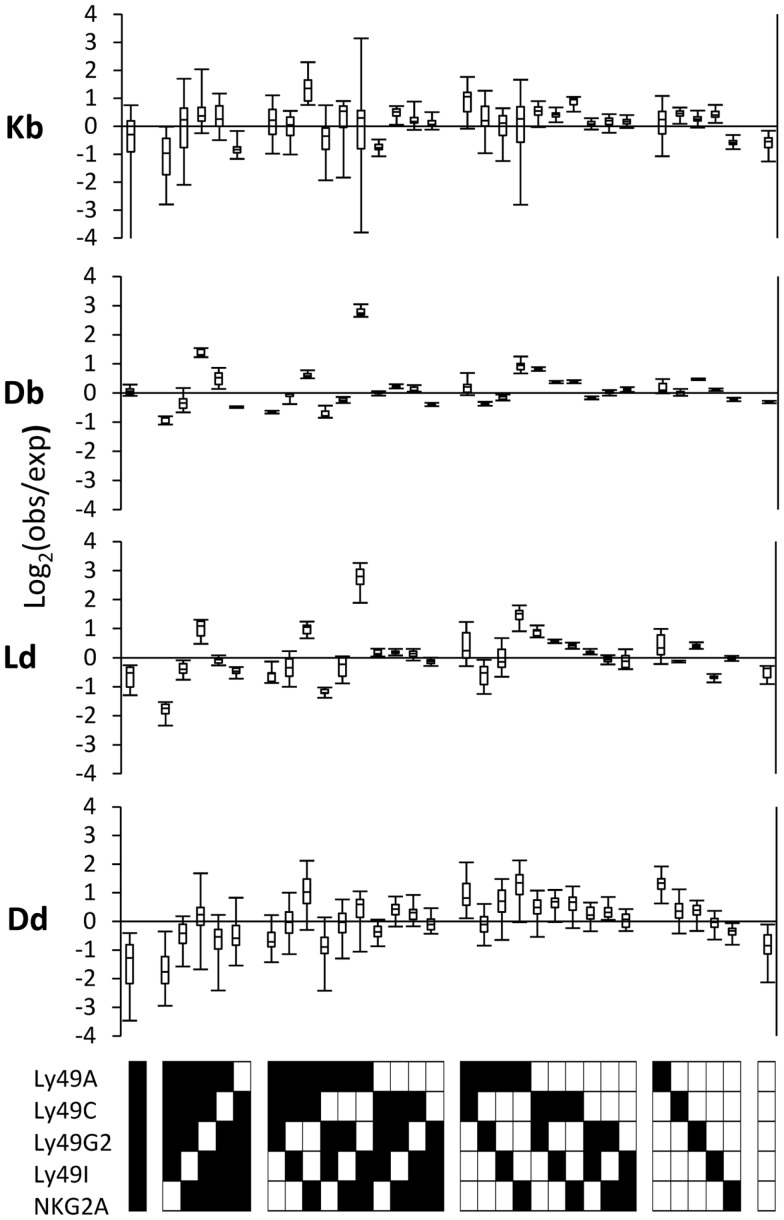
**MHC-dependent deviations from the product rule in single MHC mice**. The deviations from the product rule of 37 K^b^ mice, 6 D^b^ mice, 6 L^d^ mice, and 34 D^d^ mice, after subtracting, for each mouse and each receptor combination, the average deviation observed in 43 MHC mice for the same combination.

To test whether different MHC class I alleles produced distinct changes of the repertoire, we compared four strains expressing single MHC class I alleles (Johansson et al., 2005, 2009) and subtracted, from their PR deviations, the average PR deviations of each receptor combination in MHC-deficient mice (Figure 4). NK cells lacking all inhibitory receptors were fewer than expected in all strains, suggesting that this is a common deviation induced by all MHC class I alleles. Secondly, H2D^d^ promoted a deviation of the Ly49A single receptor-expressing subsets to an extent not observed by the other alleles (Figure 4), which is in line with our previously published results (Brodin et al., 2012). On the same note, Ly49C single-positive NK cells were more prevalent than expected in H2K^b^ mice compared to H2D^b^ mice, in line with the identification of H2K^b^ as a strong Ly49C ligand in B6 mice (Hanke et al., 1999). Thirdly, H2D^b^ and H2L^d^, which are very similar in terms of sequences and expression properties, produced a nearly identical perturbation pattern with several deviations, including a distinct over-representation of one particular subset expressing the combination of Ly49A, Ly49I, and NKG2A (Figure 4, middle two panels). Finally, we noted that the MHC class I allele with the broadest specificity of the ones tested, H2D^d^ (Hanke et al., 1999; Johansson et al., 2005, 2009) produced a deviation pattern that was most similar to the one seen in B6 mice, where two alleles exert an educating impact on the NK cell repertoire.

### The average human repertoire is highly diverse

**Figure 5:**
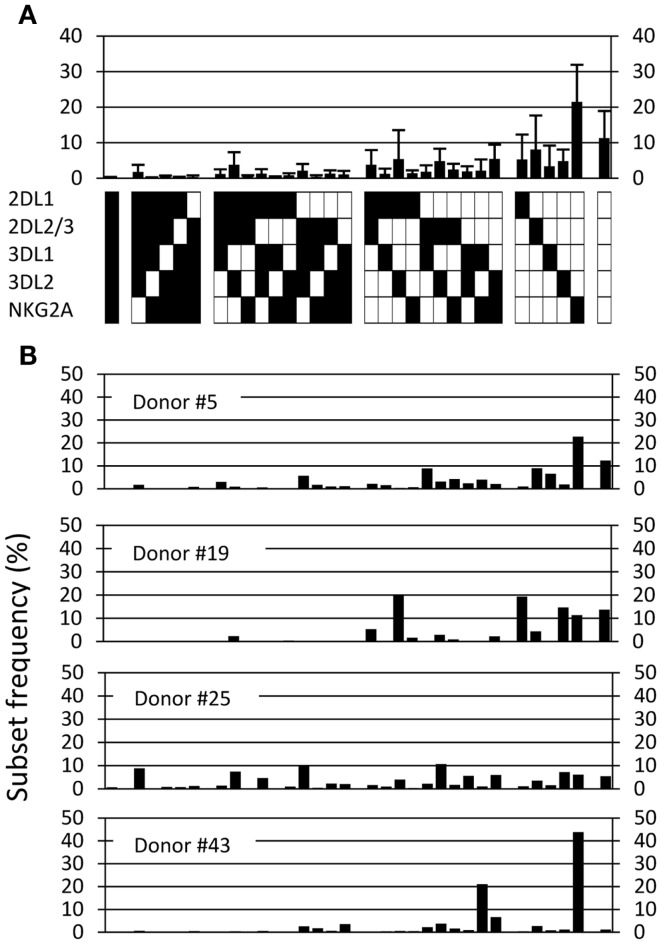
**The human repertoire is highly diverse**. **(A)** The observed frequency of each of 32 possible combinations, averaged for 44 haplotype A donors. Error bars indicate the standard deviation. **(B)** The observed repertoires in four individual haplotype A donors.

We next performed a similar repertoire analysis in a cohort of 44 healthy donors that were homozygous for the group A KIR haplotype (Fauriat et al., 2008; Andersson et al., 2009). Superficially, the average human KIR repertoire was quite similar to the mouse Ly49 repertoire, with more than 10% of NK cells expressing no inhibitory receptor, and an overall larger fraction of subsets expressing one or two inhibitory receptors compared to subsets expressing more than three receptors (Figure 5A). As expected given the genetic heterogeneity of outbred humans, we observed high variation between individuals, resulting in large standard deviations. The nature of the variation became clear when four individual human repertoires, expressing an identical setup of KIR ligands (not shown), were plotted side-by-side (Figure 5B). Among these four donors, the first (donor #5) had an inhibitory receptor repertoire that resembled that of B6 mice in terms of the distribution of subsets expressing distinct receptor combinations. The other three (donors # 19, 25, 43) were different, and showed either a more restricted expression pattern (donors #19 and 43) or a more equal distribution of subsets (donor #25). Altogether, these data support previous results showing substantial KIR ligand-independent variations in the inhibitory receptor repertoires in humans (Gumperz et al., 1996; Shilling et al., 2002; Andersson et al., 2009).

### Patterns of product rule deviations reveal differences between mouse and human repertoires

**Figure 6:**
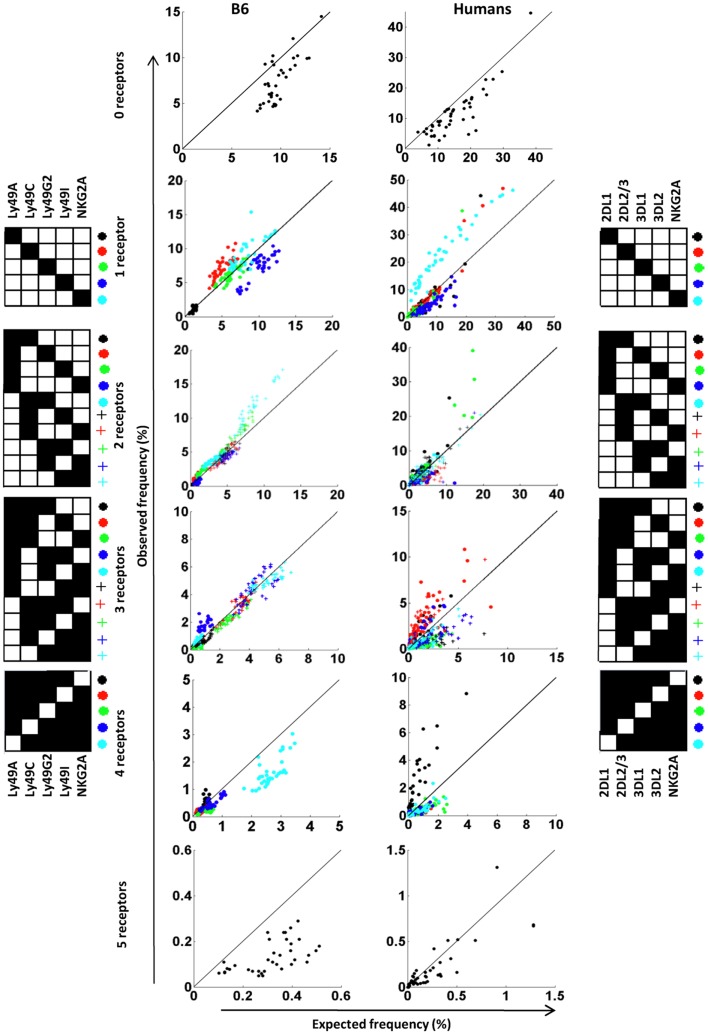
**Deviations from the product rule in B6 mice and in humans**. The observed frequency of NK cells co-expressing zero to five receptor combinations are plotted against the expected frequency, in 37 B6 mice (left panel) and 44 A haplotype human donors (right panel). A perfect fit to the product rule is shown as a straight line. Each symbol indicates a specific receptor combination, as shown on the panels beside the figures.

Because PR calculations are performed similarly in mice and humans, we analyzed the repertories in these species side-by-side by grouping subsets according to the number of receptors they express. Observed and expected frequencies of each subset were then visualized in a dot plot. Four observations were made in this comparison. First, NK cells devoid of inhibitory receptors were less frequent than expected in both B6 mice and in outbred humans (Figure 6, upper two plots), suggesting that under-representation of such NK cells is a general rule. Secondly, for NK cells expressing single inhibitory receptors, NKG2A-expressing NK cells were strongly over-represented in humans (Valiante et al., 1997; Fauriat et al., 2008; Yawata et al., 2008) while no deviation of this subset was seen in B6 mice. Linked to this notion, for subsets expressing three to five receptors, the presence or absence of NKG2A determined whether or not the subset was over- or under-represented relative to the expected frequency (Figure 6). A similar role for the NKG2A receptor was not observed in B6 mice. Thirdly, in humans, three donors showed a strong over-representation of KIR2DL2/3-positive NK cells (red dots) and one donor showed a similar over-representation of KIR2DL1-positive NK cells (black dots). It is tempting to speculate that these deviations resulted from a CMV-driven and KIR ligand-dependent skewing of the repertoire, as we recently noted in a much larger cohort of donors (Beziat et al., 2013). However, this remains a speculation since serological data regarding previous exposure to CMV is lacking in this cohort. Finally, subsets expressing all five receptors were strongly under-represented in B6 mice but not in humans. Thus, our comparison, despite suffering from some drawbacks (see Discussion) revealed similarities as well as differences between B6 mice and humans in the formation of inhibitory receptor repertoires.

## Discussion

A distribution of cellular subsets based on variegated expression of inhibitory receptors is a unique and intriguing property of human and murine NK cells (Moesta and Parham, 2012). To understand why this expression pattern has evolved, it is important to consider that the number of different inhibitory receptors an NK cell expresses determine its function under certain circumstances (Hanke and Raulet, 2001; Yu et al., 2007). For example, to elicit a “missing self” response, an NK cell expressing two different inhibitory KIR for self MHC class I will require downregulation of the HLA ligands for both of those. In contrast, an NK cell carrying a single Ly49 or KIR receptor will respond to targets missing single HLA class I alleles and will thus be sensitive to specific alterations in HLA expression.

A stochastic and independent expression pattern of each KIR or Ly49 gene represent one solution to generate NK cells with restricted numbers of Ly49 or KIR receptors (Cichocki et al., 2011; Gays et al., 2011). In this process, the development of NK cells expressing several or zero receptors is unavoidable, however, leading to the formation of subsets that might either be constantly inhibited by self MHC (many self receptors), or potentially autoreactive as they are not inhibited by self (no receptors). Subsets lacking inhibitory receptors for self may cause harm, but there are mechanisms to make them tolerant (Fernandez et al., 2005; Johansson et al., 2005; Kim et al., 2005; Anfossi et al., 2006). In this general respect, NK cell education bears similarities to T cell selection, in which harmful T cells may arise that are kept in control by complementary mechanisms. Which NK cell functions *in vivo* that are controlled by the “missing self” principle is unknown (Orr et al., 2010) and this question represents one area of intensive research.

Because of the genetic homogeneity and standardized housing conditions of inbred mice, genetic factors that have been implicated in the control of KIR expression in humans (such as promoter use, gene copy number, allelic variants, and infection history) are neutralized. It was therefore surprising that the observed repertoires showed a rather large variation between individual mice. This variation was caused by a combination of inter-experimental variation (which was largest) and intra-experimental variation. It is not known if this is caused by technical aspects of the analysis, or if individual mice differ from each other due to biological fluctuations in receptor expression. Longitudinal analysis of NK cell repertoires by repeated analyses of individual mice may give some clues to this question.

The significant and consistent deviations from the PR in mice deficient for MHC class I heavy chains was unexpected. One explanation for these deviations is that the expression probability of the various genes in the NKC are not independent, and that the expression of one gene in NKC may affect the probability of another gene to be expressed. Such dependencies in the acquisition of multiple inhibitory KIRs has been suggested in humans to explain the extensive PR deviations observed in NK cells expressing three and four inhibitory KIRs (Gardiner et al., 2001; Andersson et al., 2009). Local access to transcription factors or simultaneous demethylation of multiple promoter regions, could also play a role. It is also possible that ligands for inhibitory receptors other than classical MHC class I molecules may affect receptor skewing. Such ligands can at least influence licensing of subsets, as shown recently for the non-classical H2-M3 gene (Andrews et al., 2012). Another possibility is that there are biochemical dependencies between inhibitory receptors that affect their transport or coexpression at the cell surface.

A remaining question, especially in humans, is whether the repertoire is determined only by genetically controlled mechanisms acting intrinsically within the NK cell, or if there are aspects of the repertoire that are subject to selection to optimize it for missing self recognition. For example, are there processes that positively select certain subsets and negatively select other subsets, depending on the MHC class I setup? When testing this question, MHC class I-independent skewing effects must be taken into account. In our study, we decided to subtract the deviations in MHC-deficient mice to allow a better resolution of the MHC class I-controlled repertoire in B6 mice. By doing so, we were able to draw some general conclusions, as follows. First, NK cell subsets that express no inhibitory receptors (out of those studied) were under-represented in B6 mice. These are potentially harmful as they may not be inhibited by MHC class I-expressing self cells. Their under-representation adds an additional layer of protection to the hypo-responsiveness that such cells exhibit in “missing self” contexts (Fernandez et al., 2005; Kim et al., 2005; Anfossi et al., 2006).

Secondly, a large fraction of subsets were more frequent than expected in B6 mice, including all subsets expressing two inhibitory receptors, and most (but not all) subsets expressing three receptors. This finding suggests that MHC class I-mediated effects are broader than previously thought. Thirdly, NK cells expressing one known self receptor in isolation, Ly49C, Ly49G2, and NKG2A, showed different skewing patterns. This latter finding speaks against our previously proposed notion of positive selection as a general mechanism to enrich for NK cell subsets expressing a single self receptor. Still, data on the selective accumulation of Ly49C in the presence of H2K^b^ (this paper) and Ly49A in the presence of H2D^d^ (this paper and Brodin et al., 2012) suggest that positive selection may operate for some such subsets, even if other subsets with expression of a single self receptor behave differently. One interesting property of Ly49A and Ly49C, but not Ly49I, that comes to mind in this respect is their abilities to bind their respective MHC class I ligands in *cis* (Kase et al., 1998; Doucey et al., 2004; Andersson et al., 2007; Back et al., 2007; Scarpellino et al., 2007). NKG2A has not been studied in this respect. Finally, subsets expressing four or five receptors tended to be under-represented in B6 mice. Overall, the difference in repertoire composition between B6 mice and MHC-mice confirm a role for self MHC class I in shaping the repertoire. Much more work is required before we understand all alterations, in particular alterations of subsets expressing two or three receptors. At this point, we conclude that the effects of MHC class I was surprisingly broad and involved most subsets.

In humans, it has been more difficult to detect HLA class I-mediated skewing of the KIR repertoires. In fact, because random acquisition of KIRs in itself results in repertoires dominated by NK cells expressing few KIRs (most of which express at least one self-specific KIR), the existence of an intrinsic selection process has been questioned (Andersson et al., 2009). Nevertheless, some studies have detected increased frequencies of NK cells expressing receptors for HLA-C (Schonberg et al., 2011b) and HLA-Bw4 (Yawata et al., 2006) in individuals expressing ligands for these receptors. In our previous work, we failed to see a global skewing toward self (Andersson et al., 2009) that was predicted by models of selection favoring NK cells with self-specific receptors. Although it is possible that the inherent variation in KIR expression between individuals is too large to detect global effects, the finding that neonatal NK cells lack even subtle skewing toward self (Schonberg et al., 2011a), suggest that human repertoires are formed randomly and independently of HLA class I. The dichotomy between subtle skewing effects in human adults but nor in cord blood may be explained by our identification of CMV as a cofactor in HLA-driven repertoire skewings (Beziat et al., 2013).

The mechanisms underlying MHC class I-associated perturbations of the repertoire are unknown. Because all subsets in the repertoire add up to 100%, changes can be explained either by proliferation of some or death of other subsets. Alternatively, MHC class I may impose effects on the probability to express an additional receptor to the ones a developing cell already has, without effect on death or cell division. Our previous data showed that Ly49A^+^ NK cells from mice expressing the Ly49A ligand H2D^d^ responded better to IL-15 than Ly49A^+^ NK cells from MHC-deficient mice (Brodin et al., 2012), suggesting that skewing may be mediated by specific expansion of educated NK cells. As already mentioned, the fact that individual single MHC class I alleles produce specific changes in the repertoire consistent with their known specificities for Ly49 receptors, imply positive selection of at least some useful subsets as one driver for repertoire adaptations. Recent data from humans suggest a similar scenario, at least in the context of viral infection. Thus, acute and latent CMV infection is associated with a profound imprint in the human KIR repertoire caused by an expansion of NK cells expressing self-specific KIRs (Della Chiesa et al., 2012; Foley et al., 2012; Beziat et al., 2013). *In vitro*, NK cells educated by self-specific inhibitory KIRs expand specifically when stimulated by target cells expressing HLA-E or fibroblasts infected with CMV (Beziat et al., 2012, 2013; Charoudeh et al., 2013). Although it is still unclear whether the expansion of NK cells expressing self-specific KIRs is useful for the host or not, the resolution of CMV reactivation in a patient with T and B cell SCID correlated closely with the expansion and contraction of NKG2C+ NK cells expressing self-specific KIRs (Kuijpers et al., 2008).

Product rule calculations represent a tool to estimate deviations of subsets frequencies relative to the expected, assuming that individual receptors are independently expressed. To make this comparison, the algorithm must be fed the individual expression probabilities of all receptors under study. Because these probabilities are unknown, it is instead assumed from the overall frequency of a given receptor in the repertoire. While this procedure makes PR calculations possible in all individuals, it also contains a circular loop in that the overall frequency of a given receptor will be affected by deviations of other subsets, which is what PR calculations intend to measure. This caveat carries a risk of underestimating small subset deviations. On the other hand, deviations observed despite this may perhaps be more safely interpreted, even if this must still be done with caution, and with the possibility of other potentially contributing mechanisms in mind.

Exclusive calculations of product rule deviations were used to analyze an inbred mouse strain and a cohort of humans in parallel. Of note, our mouse cohort represent 37 independent observations of one single genotype while the human cohort included 44 outbred donors. General conclusions regarding the diversity of the repertoire in the population may therefore be made in humans, but not in mice. In contrast, the genetic heterogeneity of humans may hide specific effects in individuals, while the mouse analysis is stronger on that point. To conceptualize the difference, one could view the mouse data as an example of a repeated analysis of a single case in the human cohort.

A side-by-side view still gave several insights. Taking a look at the human cohort, we first concluded that NK cells lacking inhibitory receptors were under-represented. This observation is consistent with a model in which MHC class I proteins positively select cells expressing inhibitory receptors. Our data on B6 mice as well as four single MHC class I mice support this as a mechanism also in mice. The most notable feature of the human repertoire was the effects of NKG2A expression on NK cells, which was manifested as an over-representation of single-NKG2A-expressing NK cells and an under-representation of subsets expressing NKG2A together with KIR. One way to interpret this result is to postulate that the expression of NKG2A would make subsequent KIR expression less likely. In this scenario, an NK cell that express NKG2A as its first inhibitory receptor would tend to be over-represented and NK cells expressing NKG2A in association with KIR would be less frequent. Perhaps this model could also explain why NKG2A has been observed to “buffer” the repertoire in some donors (Yawata et al., 2008; Andersson et al., 2009). Thus, in cases where the probability to express NKG2A is high, most NK cells would lack KIR and express only NKG2A, while donors with a low expression probability of NKG2A would have a repertoire dominated by KIR expression (Valiante et al., 1997). Intriguingly, in B6 mice, a similar role for NKG2A was not seen in the repertoire. As alluded to, it is not known if this is a feature of the B6 background or a general property of all mouse strains.

The inverse correlation between NKG2A and KIR in the NK cell repertoire may also reflect population of cells at discrete stages of differentiation (Bjorkstrom et al., 2010; Beziat et al., 2011). NKG2A is expressed by all CD56^bright^ cells and is gradually lost during terminal NK cell differentiation within the CD56^dim^ stage, correlating with the acquisition of KIRs and expression of CD57 as well as loss of CD62L and NKp30 (Bjorkstrom et al., 2010; Juelke et al., 2010). The time frame for these events remains unknown. It is possible that cells acquire their KIR repertoire very early during development and that the original set up of expressed KIRs determine whether the cells are “allowed” to lose NKG2A during their continued differentiation.

The term “NK cell education” has been defined by many to mean the acquisition of functional competence in NK cells, resulting from interactions with MHC class I molecules. We have proposed that alterations in the inhibitory receptor repertoire may be included in a broader definition of NK cell education (Hoglund and Brodin, 2010). Our data, from this and other papers, on repertoire perturbations in single MHC class I mice and CMV-positive humans, support this view. The link between functional education and repertoire skewing, both in terms of regulation of global NK cell responses and in terms of signaling pathways involved remains to be determined. In addition, the process of NK cell education is still poorly defined in space and in time, and may involve a combination of developmental influences on immature precursors and tuning of mature NK cells in the periphery. Presently, we favor the view that MHC class I molecules educate the NK cell system by many means, one being to license them to become functional and the other being shaping the repertoire toward the expression of certain combinations of receptors. Further work in our laboratories is directed toward the understanding of how these two processes are linked.

## Conflict of Interest Statement

The authors declare that the research was conducted in the absence of any commercial or financial relationships that could be construed as a potential conflict of interest.

## References

[B1] Achour A. Persson K. Harris R. A. Sundback J. Sentman C. L. Lindqvist Y. (1998). The crystal structure of H-2Dd MHC class I complexed with the HIV-1-derived peptide P18-I10 at 2.4 A resolution: implications for T cell and NK cell recognition. Immunity 9, 199–208 10.1016/S1074-7613(00)80602-0 9729040

[B2] Andersson K. E. Williams G. S. Davis D. M. Hoglund P. (2007). Quantifying the reduction in accessibility of the inhibitory NK cell receptor Ly49A caused by binding MHC class I proteins in cis. Eur. J. Immunol. 37, 516–527 10.1002/eji.200636562 17236237

[B3] Andersson S. Fauriat C. Malmberg J. A. Ljunggren H. G. Malmberg K. J. (2009). KIR acquisition probabilities are independent of self-HLA class I ligands and increase with cellular KIR expression. Blood 114, 95–104 10.1182/blood-2009-08-239301 19304956

[B4] Andrews D. M. Sullivan L. C. Baschuk N. Chan C. J. Berry R. Cotterell C. L. (2012). Recognition of the nonclassical MHC class I molecule H2-M3 by the receptor Ly49A regulates the licensing and activation of NK cells. Nat. Immunol. 13, 1171–1177 10.1038/ni.2468 23142773 PMC3913127

[B5] Anfossi N. Andre P. Guia S. Falk C. S. Roetynck S. Stewart C. A. (2006). Human NK cell education by inhibitory receptors for MHC class I. Immunity 25, 331–342 10.1016/j.immuni.2006.06.013 16901727

[B6] Back J. Chalifour A. Scarpellino L. Held W. (2007). Stable masking by H-2Dd cis ligand limits Ly49A relocalization to the site of NK cell/target cell contact. Proc. Natl. Acad. Sci. U.S.A. 104, 3978–3983 10.1073/pnas.0607418104 17360463 PMC1820694

[B7] Beziat V. Dalgard O. Asselah T. Halfon P. Bedossa P. Boudifa A. (2012). CMV drives clonal expansion of NKG2C+ NK cells expressing self-specific KIRs in chronic hepatitis patients. Eur. J. Immunol. 42, 447–457 10.1002/eji.201141826 22105371

[B8] Beziat V. Duffy D. Quoc S. N. Le Garff-Tavernier M. Decocq J. Combadiere B. (2011). CD56brightCD16+ NK cells: a functional intermediate stage of NK cell differentiation. J. Immunol. 186, 6753–6761 10.4049/jimmunol.1100330 21555534

[B9] Beziat V. Liu L. Malmberg J. A. Ivarsson M. A. Sohlberg E. Bjorklund A. T. (2013). NK cell responses to cytomegalovirus infection lead to stable imprints in the human KIR repertoire and involve activating KIRs. Blood. (in press). 23325834 10.1182/blood-2012-10-459545 PMC3617633

[B10] Bjorkstrom N. K. Riese P. Heuts F. Andersson S. Fauriat C. Ivarsson M. A. (2010). Expression patterns of NKG2A, KIR, and CD57 define a process of CD56dim NK-cell differentiation uncoupled from NK-cell education. Blood 116, 3853–3864 10.1182/blood-2010-04-281675 20696944

[B11] Brodin P. Hoglund P. (2008). Beyond licensing and disarming: a quantitative view on NK-cell education. Eur. J. Immunol. 38, 2934–2937 10.1002/eji.200838760 18979511

[B12] Brodin P. Lakshmikanth T. Johansson S. Karre K. Hoglund P. (2009). The strength of inhibitory input during education quantitatively tunes the functional responsiveness of individual natural killer cells. Blood 113, 2434–2441 10.1182/blood-2008-05-156836 18974374

[B13] Brodin P. Lakshmikanth T. Karre K. Hoglund P. (2012). Skewing of the NK cell repertoire by MHC class I via quantitatively controlled enrichment and contraction of specific Ly49 subsets. J. Immunol. 188, 2218–2226 10.4049/jimmunol.1102801 22287714

[B14] Charoudeh H. N. Terszowski G. Czaja K. Gonzalez A. Schmitter K. Stern M. (2013). Modulation of the natural killer cell KIR repertoire by cytomegalovirus infection. Eur. J. Immunol. 43, 480–487 10.1002/eji.201242389 23161492

[B15] Chen Y. Shi Y. Cheng H. An Y. Q. Gao G. F. (2009). Structural immunology and crystallography help immunologists see the immune system in action: how T and NK cells touch their ligands. IUBMB Life 61, 579–590 10.1002/iub.208 19472182

[B16] Cichocki F. Miller J. S. Anderson S. K. (2011). Killer immunoglobulin-like receptor transcriptional regulation: a fascinating dance of multiple promoters. J. Innate Immun. 3, 242–248 10.1159/000323929 21411970 PMC3128145

[B17] Cooley S. Trachtenberg E. Bergemann T. L. Saeteurn K. Klein J. Le C. T. (2009). Donors with group B KIR haplotypes improve relapse-free survival after unrelated hematopoietic cell transplantation for acute myelogenous leukemia. Blood 113, 726–732 10.1182/blood-2008-07-171926 18945962 PMC2628378

[B18] Della Chiesa M. Falco M. Podesta M. Locatelli F. Moretta L. Frassoni F. (2012). Phenotypic and functional heterogeneity of human NK cells developing after umbilical cord blood transplantation: a role for human cytomegalovirus? Blood 119, 399–410 10.1182/blood-2011-08-372003 22096237

[B19] Doucey M. A. Scarpellino L. Zimmer J. Guillaume P. Luescher I. F. Bron C. (2004). Cis association of Ly49A with MHC class I restricts natural killer cell inhibition. Nat. Immunol. 5, 328–336 10.1038/nrg1358 14973437

[B20] Fahlen L. Lendahl U. Sentman C. L. (2001). MHC class I-Ly49 interactions shape the Ly49 repertoire on murine NK cells. J. Immunol. 166, 6585–6592 11359811 10.4049/jimmunol.166.11.6585

[B21] Fauriat C. Andersson S. Bjorklund A. T. Carlsten M. Schaffer M. Bjorkstrom N. K. (2008). Estimation of the size of the alloreactive NK cell repertoire: studies in individuals homozygous for the group A KIR haplotype. J. Immunol. 181, 6010–6019 18941190 10.4049/jimmunol.181.9.6010

[B22] Fernandez N. C. Treiner E. Vance R. E. Jamieson A. M. Lemieux S. Raulet D. H. (2005). A subset of natural killer cells achieves self-tolerance without expressing inhibitory receptors specific for self-MHC molecules. Blood 105, 4416–4423 10.1182/blood-2004-08-3156 15728129 PMC1895026

[B23] Foley B. Cooley S. Verneris M. R. Pitt M. Curtsinger J. Luo X. (2012). Cytomegalovirus reactivation after allogeneic transplantation promotes a lasting increase in educated NKG2C+ natural killer cells with potent function. Blood 119, 2665–2674 10.1182/blood-2011-10-386995 22180440 PMC3311280

[B24] Gardiner C. M. Guethlein L. A. Shilling H. G. Pando M. Carr W. H. Rajalingam R. (2001). Different NK cell surface phenotypes defined by the DX9 antibody are due to KIR3DL1 gene polymorphism. J. Immunol. 166, 2992–3001 11207248 10.4049/jimmunol.166.5.2992

[B25] Gays F. Koh A. S. Mickiewicz K. M. Aust J. G. Brooks C. G. (2011). Comprehensive analysis of transcript start sites in ly49 genes reveals an unexpected relationship with gene function and a lack of upstream promoters. PLoS ONE 6:e18475 10.1371/journal.pone.0018475 21483805 PMC3069108

[B26] Gumperz J. E. Valiante N. M. Parham P. Lanier L. L. Tyan D. (1996). Heterogeneous phenotypes of expression of the NKB1 natural killer cell class I receptor among individuals of different human histocompatibility leukocyte antigens types appear genetically regulated, but not linked to major histocompatibililty complex haplotype. J. Exp. Med. 183, 1817–1827 10.1084/jem.183.4.1817 8666938 PMC2192483

[B27] Hanke T. Raulet D. H. (2001). Cumulative inhibition of NK cells and T cells resulting from engagement of multiple inhibitory Ly49 receptors. J. Immunol. 166, 3002–3007 11207249 10.4049/jimmunol.166.5.3002

[B28] Hanke T. Takizawa H. McMahon C. W. Busch D. H. Pamer E. G. Miller J. D. (1999). Direct assessment of MHC class I binding by seven Ly49 inhibitory NK cell receptors. Immunity 11, 67–77 10.1016/S1074-7613(00)80082-5 10435580

[B29] Held W. Dorfman J. R. Wu M. F. Raulet D. H. (1996). Major histocompatibility complex class I-dependent skewing of the natural killer cell Ly49 receptor repertoire. Eur. J. Immunol. 26, 2286–2292 10.1002/eji.1830261003 8898935

[B30] Hiby S. E. Walker J. J. O’shaughnessy K. M. Redman C. W. Carrington M. Trowsdale J. (2004). Combinations of maternal KIR and fetal HLA-C genes influence the risk of preeclampsia and reproductive success. J. Exp. Med. 200, 957–965 10.1084/jem.20041214 15477349 PMC2211839

[B31] Hilton H. G. Vago L. Older Aguilar A. M. Moesta A. K. Graef T. Abi-Rached L. (2012). Mutation at positively selected positions in the binding site for HLA-C shows that KIR2DL1 is a more refined but less adaptable NK cell receptor than KIR2DL3. J. Immunol. 189, 1418–1430 10.4049/jimmunol.1100431 22772445 PMC3439511

[B32] Hoglund P. Brodin P. (2010). Current perspectives of natural killer cell education by MHC class I molecules. Nat. Rev. Immunol. 10, 724–734 10.1038/nri2835 20818413

[B33] Hoglund P. Ljunggren H. G. Ohlen C. Ahrlund-Richter L. Scangos G. Bieberich C. (1988). Natural resistance against lymphoma grafts conveyed by H-2Dd transgene to C57BL mice. J. Exp. Med. 168, 1469–1474 10.1084/jem.168.4.1469 3171481 PMC2189085

[B34] Jiang W. Johnson C. Jayaraman J. Simecek N. Noble J. Moffatt M. F. (2012). Copy number variation leads to considerable diversity for B but not A haplotypes of the human KIR genes encoding NK cell receptors. Genome Res. 22, 1845–1854 10.1101/gr.133926.111 22948769 PMC3460180

[B35] Johansson S. Johansson M. Rosmaraki E. Vahlne G. Mehr R. Salmon-Divon M. (2005). Natural killer cell education in mice with single or multiple major histocompatibility complex class I molecules. J. Exp. Med. 201, 1145–1155 10.1084/jem.20050167 15809355 PMC2213126

[B36] Johansson S. Salmon-Divon M. Johansson M. H. Pickman Y. Brodin P. Karre K. (2009). Probing natural killer cell education by Ly49 receptor expression analysis and computational modelling in single MHC class I mice. PLoS ONE 4:e6046 10.1371/journal.pone.0006046 19557128 PMC2699029

[B37] Juelke K. Killig M. Luetke-Eversloh M. Parente E. Gruen J. Morandi B. (2010). CD62L expression identifies a unique subset of polyfunctional CD56dim NK cells. Blood 116, 1299–1307 10.1182/blood-2009-11-253286 20505160

[B38] Kase A. Johansson M. H. Olsson-Alheim M. Y. Karre K. Hoglund P. (1998). External and internal calibration of the MHC class I-specific receptor Ly49A on murine natural killer cells. J. Immunol. 161, 6133–6138 9834098

[B39] Khakoo S. I. Thio C. L. Martin M. P. Brooks C. R. Gao X. Astemborski J. (2004). HLA and NK cell inhibitory receptor genes in resolving hepatitis C virus infection. Science 305, 872–874 10.1126/science.1097670 15297676

[B40] Kim S. Poursine-Laurent J. Truscott S. M. Lybarger L. Song Y. J. Yang L. (2005). Licensing of natural killer cells by host major histocompatibility complex class I molecules. Nature 436, 709–713 10.1038/nature03877 16079848

[B41] Kuijpers T. W. Baars P. A. Dantin C. Van Den Burg M. Van Lier R. A. Roosnek E. (2008). Human NK cells can control CMV infection in the absence of T cells. Blood 112, 914–915 10.1182/blood-2008-05-157354 18650467

[B42] Lemonnier F. A. (2002). The utility of H-2 class I knockout mice. Virus Res. 82, 87–90 10.1016/S0168-1702(01)00392-6 11885956

[B43] Ljunggren H. G. Karre K. (1990). In search of the ‘missing self’: MHC molecules and NK cell recognition. Immunol. Today 11, 237–244 10.1016/0167-5699(90)90097-S 2201309

[B44] Makrigiannis A. P. Anderson S. K. (2000). Ly49 gene expression in different inbred mouse strains. Immunol. Res. 21, 39–47 10.1385/IR:21:1:39 10803882

[B45] Martin M. P. Gao X. Lee J. H. Nelson G. W. Detels R. Goedert J. J. (2002). Epistatic interaction between KIR3DS1 and HLA-B delays the progression to AIDS. Nat. Genet. 31, 429–434 12134147 10.1038/ng934

[B46] Mehr R. Sternberg-Simon M. Michaeli M. Pickman Y. (2012). Models and methods for analysis of lymphocyte repertoire generation, development, selection and evolution. Immunol. Lett. 148, 11–22 10.1016/j.imlet.2012.08.002 22902400

[B47] Moesta A. K. Parham P. (2012). Diverse functionality among human NK cell receptors for the C1 epitope of HLA-C: KIR2DS2, KIR2DL2, and KIR2DL3. Front. Immunol. 3:336 10.3389/fimmu.2012.00336 23189078 PMC3504360

[B48] Moretta L. Moretta A. (2004). Killer immunoglobulin-like receptors. Curr. Opin. Immunol. 16, 626–633 10.1016/j.coi.2004.07.010 15342010

[B49] Orr M. T. Murphy W. J. Lanier L. L. (2010). ‘Unlicensed’ natural killer cells dominate the response to cytomegalovirus infection. Nat. Immunol. 11, 321–327 10.1038/ni.1849 20190757 PMC2842453

[B50] Pascal V. Stulberg M. J. Anderson S. K. (2006). Regulation of class I major histocompatibility complex receptor expression in natural killer cells: one promoter is not enough! Immunol. Rev. 214, 9–21 10.1111/j.1600-065X.2006.00452.x 17100872

[B51] Patel R. Belanger S. Tai L. H. Troke A. D. Makrigiannis A. P. (2010). Effect of Ly49 haplotype variance on NK cell function and education. J. Immunol. 185, 4783–4792 10.4049/jimmunol.1001287 20855875

[B52] Pelak K. Need A. C. Fellay J. Shianna K. V. Feng S. Urban T. J. (2011). Copy number variation of KIR genes influences HIV-1 control. PLoS Biol. 9:e1001208 10.1371/journal.pbio.1001208 22140359 PMC3226550

[B53] Perarnau B. Saron M. F. San Martin B. R. Bervas N. Ong H. Soloski M. J. (1999). Single H2Kb, H2Db and double H2KbDb knockout mice: peripheral CD8+ T cell repertoire and anti-lymphocytic choriomeningitis virus cytolytic responses. Eur. J. Immunol. 29, 1243–1252 10.1002/(SICI)1521-4141(199904)29:04<1243::AID-IMMU1243>3.0.CO;2-A 10229092

[B54] Ruggeri L. Capanni M. Urbani E. Perruccio K. Shlomchik W. D. Tosti A. (2002). Effectiveness of donor natural killer cell alloreactivity in mismatched hematopoietic transplants. Science 295, 2097–2100 10.1126/science.1068440 11896281

[B55] Salcedo M. Andersson M. Lemieux S. Van Kaer L. Chambers B. J. Ljunggren H. G. (1998). Fine tuning of natural killer cell specificity and maintenance of self tolerance in MHC class I-deficient mice. Eur. J. Immunol. 28, 1315–1321 10.1002/(SICI)1521-4141(199812)28:12<4356::AID-IMMU4356>3.3.CO;2-8 9565371

[B56] Scarpellino L. Oeschger F. Guillaume P. Coudert J. D. Levy F. Leclercq G. (2007). Interactions of Ly49 family receptors with MHC class I ligands in trans and cis. J. Immunol. 178, 1277–1284 17237373 10.4049/jimmunol.178.3.1277

[B57] Schonberg K. Fischer J. C. Kogler G. Uhrberg M. (2011a). Neonatal NK-cell repertoires are functionally, but not structurally, biased toward recognition of self HLA class I. Blood 117, 5152–5156 10.1182/blood-2010-03-273656 21415265

[B58] Schonberg K. Sribar M. Enczmann J. Fischer J. C. Uhrberg M. (2011b). Analyses of HLA-C-specific KIR repertoires in donors with group A and B haplotypes suggest a ligand-instructed model of NK cell receptor acquisition. Blood 117, 98–107 10.1182/blood-2011-02-334441 20935255

[B59] Shilling H. G. Young N. Guethlein L. A. Cheng N. W. Gardiner C. M. Tyan D. (2002). Genetic control of human NK cell repertoire. J. Immunol. 169, 239–247 12077250 10.4049/jimmunol.169.1.239

[B60] Uhrberg M. (2005). Shaping the human NK cell repertoire: an epigenetic glance at KIR gene regulation. Mol. Immunol. 42, 471–475 10.1016/j.molimm.2004.07.029 15607801

[B61] Valiante N. M. Uhrberg M. Shilling H. G. Lienert-Weidenbach K. Arnett K. L. D’andrea A. (1997). Functionally and structurally distinct NK cell receptor repertoires in the peripheral blood of two human donors. Immunity 7, 739–751 10.1016/S1074-7613(00)80393-3 9430220

[B62] Venstrom J. M. Pittari G. Gooley T. A. Chewning J. H. Spellman S. Haagenson M. (2012). HLA-C-dependent prevention of leukemia relapse by donor activating KIR2DS1. N. Engl. J. Med. 367, 805–816 10.1056/NEJMoa1200503 22931314 PMC3767478

[B63] Yawata M. Yawata N. Draghi M. Little A. M. Partheniou F. Parham P. (2006). Roles for HLA and KIR polymorphisms in natural killer cell repertoire selection and modulation of effector function. J. Exp. Med. 203, 633–645 10.1084/jem.20051884 16533882 PMC2118260

[B64] Yawata M. Yawata N. Draghi M. Partheniou F. Little A. M. Parham P. (2008). MHC class I-specific inhibitory receptors and their ligands structure diverse human NK-cell repertoires toward a balance of missing self-response. Blood 112, 2369–2380 10.1182/blood-2008-03-143727 18583565 PMC2532809

[B65] Yu J. Heller G. Chewning J. Kim S. Yokoyama W. M. Hsu K. C. (2007). Hierarchy of the human natural killer cell response is determined by class and quantity of inhibitory receptors for self-HLA-B and HLA-C ligands. J. Immunol. 179, 5977–5989 17947671 10.4049/jimmunol.179.9.5977

